# The use of experimental vignette studies to identify drivers of variations in the delivery of health care: a scoping review

**DOI:** 10.1186/s12874-021-01247-4

**Published:** 2021-04-22

**Authors:** Jessica Sheringham, Isla Kuhn, Jenni Burt

**Affiliations:** 1 Department of Applied Health Research, UCL, 1-19 Torrington Place, London, WC1E 7HB UK; 2 The Healthcare Improvement Studies (THIS) Institute, University of Cambridge, Cambridge Biomedical Campus, Clifford Allbutt Building, Cambridge, CB2 0AH UK

**Keywords:** Vignettes, Healthcare variation, Inequalities

## Abstract

**Background:**

Identifying how unwarranted variations in healthcare delivery arise is challenging. Experimental vignette studies can help, by isolating and manipulating potential drivers of differences in care. There is a lack of methodological and practical guidance on how to design and conduct these studies robustly. The aim of this study was to locate, methodologically assess, and synthesise the contribution of experimental vignette studies to the identification of drivers of unwarranted variations in healthcare delivery.

**Methods:**

We used a scoping review approach. We searched MEDLINE, Embase, Web of Science and CINAHL databases (2007–2019) using terms relating to vignettes and variations in healthcare. We screened title/abstracts and full text to identify studies using experimental vignettes to examine drivers of variations in healthcare delivery. Included papers were assessed against a methodological framework synthesised from vignette study design recommendations within and beyond healthcare.

**Results:**

We located 21 eligible studies. Study participants were almost exclusively clinicians (18/21). Vignettes were delivered via text (*n* = 6), pictures (n = 6), video (n = 6) or interactively, using face-to-face, telephone or online simulated consultations (*n* = 3). Few studies evaluated the credibility of vignettes, and many had flaws in their wider study design. Ten were of good methodological quality. Studies contributed to understanding variations in care, most commonly by testing hypotheses that could not be examined directly using real patients.

**Conclusions:**

Experimental vignette studies can be an important methodological tool for identifying how unwarranted variations in care can arise. Flaws in study design or conduct can limit their credibility or produce biased results. Their full potential has yet to be realised.

**Supplementary Information:**

The online version contains supplementary material available at 10.1186/s12874-021-01247-4.

## Introduction

Unwarranted variations in the delivery of health care are widespread [1, 2]. These variations have manifested in systematically poorer quality or lower availability of care for patients for reasons including their gender, age, ethnicity, and socioeconomic circumstances [3]. Examples of such inequalities include patients of Hispanic or South Asian ethnic backgrounds reporting poorer experience of their doctors than majority white patients in the USA and UK [4–7], and delays in cancer diagnosis (associated with poorer survival) being reported more frequently for older patients and patients in adverse socioeconomic circumstances compared to younger and majority white patients in the UK [8, 9]. Evidence on how such variations arise and persist is required to inform improvement efforts. Proposed drivers of variations in the delivery of care include individual healthcare provider perceptions or behaviours – such as the presence of implicit bias [10] – as well as variations in patient expectations or behaviours [11]. Differences in how decisions are reached as providers and patients interact may also contribute to persistent variations in care [12]. These explanations are widely proposed in many areas where variations are identified, but robust evidence often remains lacking or inconclusive [13, 14]. Obtaining actionable insights into the judgements, activities and behaviours of individuals within health care systems is challenging. It is even more challenging when the situations under scrutiny are rare, occur in complex settings, or raise difficult ethical questions [15]. Experimental vignette studies offer one methodological approach to tackling this challenge.

A vignette is a short, carefully constructed depiction of a person, object, or situation, representing a systematic combination of characteristics [16]. First used in ethnographic fieldwork to prompt informants for more detailed reflection [17], hypothetical scenarios were subsequently adopted by experimental psychologists to examine cognitive processes [18, 19]. Vignette approaches have since been taken up in diverse fields including social science [15, 20, 21], organisational research [22, 23], applied and social psychology [24], business ethics [25], information studies [26], and nursing research [27].

In experimental vignette studies, vignettes are used to explore participants’ attitudes, judgements, beliefs, emotions, knowledge or likely behaviours by presenting a series of hypothetical yet realistic scenarios across which key variables have been intentionally modified whilst the remaining content of the vignette is kept constant [22, 26]. Such studies seek to generate inferences about cause-and-effect relationships by considering the nature of each vignette, and participants’ subsequent responses to these vignettes [28, 29]. Vignettes themselves may be presented using a variety of modalities, including text, pictures, video or by using actors in simulated or real clinical environments. Studies are often factorial in design, with vignettes created to represent all possible combinations of pre-defined factors of interest, and a random sample of vignettes subsequently presented to each participant [18, 27, 30]. Experimental vignette studies provide a ‘hybrid’ approach between conventional surveys and observations of real-life practice. The intentional manipulation of vignettes in experimental designs to compare the causal effects of variables enhances internal validity, whilst the survey sampling approaches available to researchers conducting vignette-based studies enhances external validity [16, 22, 31].

Opponents to vignette studies commonly note that they are not studying real life [26, 32]. Several validation studies have examined how vignettes perform against alternative methods of assessing the delivery of care, often using medical records and standardised patients as comparators [33–35]. Whilst each method inevitably has strengths and weaknesses, well designed vignette studies may have advantages in certain scenarios. Health care professionals’ choices of care in clinical vignettes have been found to reflect their stated intentions and behaviours more closely than data extracted from medical records or from recordings of real consultations [33–35]. Biases or inaccuracies may arise from observations of actual clinical practice in a number of ways. For example, evidence suggests that physicians may under-report clinical activities within medical records, possibly due to time constraints [33]. Additionally, key actions can be missed in recording doctor-patient consultations; body language is omitted from analyses of audio recordings, whilst off-camera events are missed in video recordings [36, 37]. As a result, observational studies alone may not provide sufficient depth of evidence to inform successful efforts to reduce variations in care; experimental vignette studies offer an alternative lens through which to identify key drivers of variations.

In our experience of conducting experimental vignette studies, there is a lack of methodological and practical guidance available on how to design and conduct these studies robustly. Unlike other study types, there is no universal checklist to ensure vignette studies are understandable, transparent and of high quality [38]. The aim of this scoping review was to locate, methodologically evaluate, and synthesise the contribution of experimental vignette studies that seek to identify drivers of unwarranted variations in the delivery of healthcare. In doing so, we hope to provide an overview of how to do such studies well, and what we can learn from them.

## Methods

We conducted a scoping review in accordance with PRISMA-ScR guidelines [39].

### Eligibility criteria

We aimed to locate primary empirical studies that used an experimental vignette design to examine drivers of variation in the delivery of healthcare. The review focused on drivers of variation and therefore excluded those that only sought to describe variations, as the measurement of variations is feasible using records or observation of real healthcare delivery See supplementary file 1 for full inclusion and exclusion criteria.

### Information sources and search strategy

The search strategy was developed in collaboration with an experienced information specialist (IK), and used text words and synonyms for vignettes and variations in healthcare (supplementary file 1). The following databases were searched from January 2007 to April 2019: MEDLINE (via Ovid), Embase (via Ovid), Web of Science, and CINAHL (via EBSCO). The search was limited to 2007 because the majority of methodological reviews of vignettes were published since this date (see supplementary file 2). The search strategy was developed in MEDLINE and adapted for other databases as appropriate.

### Study selection

We used a phased approach to title/abstract screening. First, an automated search of key words in titles and abstracts was undertaken using Stata15 [40] to exclude studies that were clearly of no relevance (e.g. studies published in planetary journals). We undertook manual checks of automated Stata screening exclusions to refine terms (for example, initially terms connected with education were used to exclude studies on students but removed when they were identified as excluding papers referring to qualified physicians). Next, JS manually screened the remaining titles and abstracts to exclude papers that did not examine healthcare variations using vignettes, or that measured rather than sought to identify drivers of variation. JB double screened 10% of the full sample to confirm accuracy and clarify inclusion criteria. Inter-rater agreement was assessed using Cohen’s kappa for a subset of papers. Prior to consensus discussions, Kappa was 66%, which can be interpreted as moderate agreement [41].

Full-text screening was conducted by JS with 10% double screening by JB. For both title/abstract and full-text screening, all differences were resolved by discussion.

### Data extraction

Data were extracted by JS, with 10% double extraction by JB, using a tool developed and piloted for the purposes of this study, covering setting, respondents, healthcare setting, medical condition under scrutiny, patient characteristics, drivers of variation under scrutiny, and vignette modality.

### Methodological assessment

There is no existing standardised approach to evaluating the robustness of experimental vignette studies. We therefore conducted a review of methodological reviews of vignette studies within and beyond healthcare. Synthesising insights from all included methodological papers [26, 32, 42–46], we developed a framework to appraise the design and conduct of experimental vignette studies within this review: see supplementary file 2 for full details of this review of reviews and the framework development.

Within this framework, we identified factors considered important in maximising internal and external validity of experimental vignette studies in two broad areas: (A) the design and description of vignettes used, and (B) the wider study design and methods within which vignettes are employed, as outlined below (Table 1) [46, 50, 52].

**Table 1:** Methodological framework for assessment of experimental vignette studies

A. Vignette design	
1. Credibility	• The degree to which vignettes credibly represent critical aspects of a clinical scenario or patient to potential participants is crucial to the success of an experimental vignette study [37]. • Lens model approaches (studies which compare optimal versus actual decisions in a given situation, originally developed by Brunswick in 1950) have demonstrated empirically that the decision-making performance of participants is improved when situations are realistic [47]. • Basing vignettes on real-life data, clinical expertise, and existing guidelines are recommended ways of enhancing credibility [26, 45, 46].
2. Number	• Presenting participants with more than one vignette enables examination of variations in judgement within individuals as well as between them – that is, the extent to which each participant is differentially influenced by each experimental factor in making their decisions. Where this is required to address study aims, for example, in vignette approaches based on the lens model [48], it is typically recommended that there are at least five different representations for each experimental factor. • Additional considerations are needed when several vignettes are used,, such as controlling for the order in which vignettes are presented and taking account of clustering within individuals in the analysis (see: wider study design).
3. Variability	• Developing or using a number of different representations of each experimental factor may increase study generalisability, by reducing the possibility that idiosyncrasies in one particular representation are responsible for findings. For example, using one female and one male actor in video vignettes may lead not to participants responding to the constructs of gender, but to *that* particular female or *that* particular male. • Where participants do view more than one vignette, analysis must account for clustering of vignettes by respondent, to avoid over-estimating the statistical significance of any effect [49].
4. Mode	• The mode through which vignettes are delivered has an important influence on the research question an experimental vignette study can answer. • Vignette mode has historically been textual only, with participants presented with a written scenario. Text-based vignettes may constrain not just the information the respondent is given, but how this information is framed. • More recently the use of pictures, videos, actors, and interactive environments have been developed [22, 46]. • Pictorial modes are particularly suited to examination of characteristics, such as ethnicity, where visual representation removes the need for explicit statement (and prior framing) of the characteristic. • Studies using video vignettes extend this still further by enabling participants to form judgements on body language and speech patterns in addition to visual cues. • Interactive formats, such as unannounced standardised patients or virtual reality set-ups, have the potential to mimic real delivery which enables exploration of how inequalities may unfold *during* a clinical encounter, through enabling explorations of variations in the information that clinical participants elicit from patients or in both parties’ non-verbal communication. Such approaches are more complex to construct and more costly to develop than static vignette formats, which may limit their feasibility.
5. Evaluation	• Evaluation of vignettes’ face validity – during vignette construction and once data are collected – is key to understanding the validity of findings in studies using vignettes. • Thinking through in advance what is needed to make particular vignettes ‘successful’ for their target audience will guide the nature of and approach to evaluation. • Options include assessment by an expert panel, feedback from participants, or comparing responses to the vignettes to an additional data source such as clinical data [26, 46].
6. Description	• Readers of vignette study papers need to be able to form their own judgments of vignette credibility. An entire vignette should be provided to enable them to do so.
B. Wider study design	
1. Concealment	• When investigating unwarranted variations in care, it is important to conceal the purpose of such studies, given that few people will volunteer behaviours or attitudes that they recognise as poor or biased. • If the study’s purpose is not adequately masked it can bias results, even with carefully constructed vignettes [31]. Participants may learn of the study purpose directly (from study information shared at recruitment) but also may infer it indirectly, through other cues in study materials (e.g. funder’s name), or pre-specified responses that prime participants to consider certain answers.
2. Realism	• External validity of vignette studies is enhanced when studies are conducted in a setting as close as possible to the natural ecology of decision-making [47, 50]. • The generalisability of studies to investigate unwarranted variation in healthcare may be improved by collecting data in a setting that mimics key aspects of clinical settings, whether that be the actual environment, other inclusion of features such as the imposition of time constraints.
3. Sampling & response	• The representativeness of any survey rests on sampling, coverage, and nonresponse. • This is particularly important for studies of healthcare variations, where a biased sample or responses – for physician or patient participants – may lead to over- or under-estimation of variations. • Studies need to justify their sample design, sample size, approach to recruitment, response and completion rates, and reasons for excluding data [51]. • The implications of low or biased responses should be considered.
4. Analysis	• Experimental vignette studies are often complex in how data are structured. Analysis must appropriately account for hierarchies within the data [22].

A. Vignette design

Six key considerations were identified as important in the construction and use of robust vignettes: vignette credibility, number, variability, mode, evaluation, and description. These are described in more detail in Table 1. We use the term *vignette* to refer to the overall description or depiction of each situation as presented to the participants. Within each vignette, *experimental factor/s* represent the variable/s of interest which have been intentionally modified and manipulated (such as gender or ethnicity); the *representation of experimental factor/s* refers to the varying ways in which each experimental factor is represented across the vignettes (e.g. the multiple ways in which ethnicity or gender have been presented to the participant).

B. Wider study design

Four considerations were identified as important in the overall design of experimental vignette studies: concealment, realism, sampling and response rates, and analysis (Table 1). In almost all cases, experimental vignette studies are a form of survey, and thus principles of good survey design (including standards for good questionnaire design) should be followed.

We applied this framework to all included studies to appraise the way in which they were conducted. We generated a scoring system to reflect how well studies had met eight of the ten methodological considerations. For four considerations (vignette credibility, evaluation, description and study analysis) the scores primarily reflected the extent to which sufficient methodological detail was provided. For two considerations (vignette variability and study realism) the score primarily reflected whether optimal choice in the design of the study was made. For two considerations in the wider study design (concealment and sampling/response), the scores reflected both provision of methodological detail and the quality of study execution. The sampling/response consideration was weighted most heavily in the scoring system (maximum of 6 marks) because we judged it of key importance to the credibility and validity of studies seeking to report on inequalities. Two considerations were not given a score; mode of vignette delivery and whether multiple vignettes were provided. Both these considerations - while important for researchers to consider when designing vignettes – are not, intrinsically, markers of quality.

Adding up assessments across each methodological consideration, studies were then assigned to one of three groups: good, moderate or low overall methodological quality (see Table 3 and supplementary file 2 for full details of categorisation). The cut-offs were agreed by JB and JS in part determined by their overall score and in part determined by their performance on key considerations. Studies were considered moderate rather than high quality when overall their design and reporting was good enough overall but where there were significant flaws in at least one dimension. The distinction between moderate and low quality was made where we judged studies to be too flawed to inform wider understanding of healthcare inequalities.

### Data synthesis

Studies were synthesised narratively, paying particular attention to how studies yielded insights into variations in healthcare delivery. We excluded studies judged to be of low methodological quality from this synthesis.

### Registration

As a methodological scoping review, the study was not eligible to be registered on PROSPERO.

## Results

### Study selection and characteristics

**Fig. 1:**
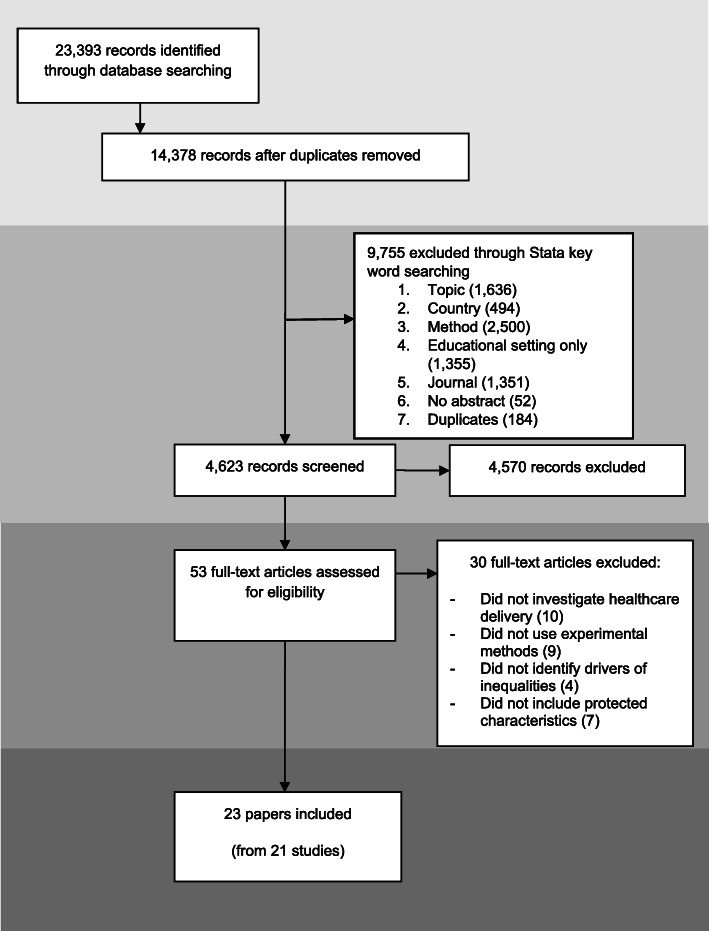
PRISMA Flow diagram

We identified 23 papers and 21 unique studies for inclusion within the review (see PRISMA flowchart, Figure 1). Most studies related to primary care settings (see Table 2 for details). Studies were most frequently based in the USA (n=14), with England (n=2), Portugal (n=1), Sweden (n=1), the Netherlands (1), France (1) and multi-country settings (n=3) also represented. Vignette participants were almost exclusively healthcare providers (20/23), predominantly doctors (n=14). Only three studies examined public perspectives on healthcare delivery [58, 62, 74].

**Table 2:** Included studies – descriptive characteristics and main findings

Study	Research question	Geographical setting	Healthcare setting	Participants	Process or decision	Patient characteristics	Possible drivers of variation	Condition	Findings
Adams et al, 2014 [53]	Identification of mechanisms driving differential diagnoses and disparities that are common to black and white people in both countries; examination ofbetween-country variations due to cultural and health care system differences	UK and USA	Primary care	Physician	Diagnosis, referral, prescription	Ethnicity (black, white)	Information processing, patient cues, knowledge used, healthcare system.	Depression	There was little bias in doctors' decisions overall. UK doctors had greater clinical uncertainty in diagnosing depression amongst black than white patients . Doctors focused more on black patients' physical than psychological symptoms and more often tended to identify endocrine problems.
Begeer et al, 2008 [54]	Whether ethnic background influences the likelihood of pediatricians’ references to Autism when using clinical judgments versus ratings of explicit diagnostic categories	Netherlands	Child health setting	Physician	Diagnosis	Ethnicity (Dutch vs Moroccan or Turkish)	Spontaneous vs prompted likelihood; physician characteristics	Autism	Spontaneous clinical judgements resulted in ethnic bias; this bias disappeared when doctors were prompted to consider autism.
Bernardes et al, 2013 [55]	Whether physician sex moderates the effects of patient (distressed) pain behaviours and diagnostic evidence of pathology on treatment prescriptions and referrals; explore the mediating role of pain credibility judgments and psychological attributions on these effects	Portugal	Primary care	Physician	Referral, prescription, assessment	Gender	Physician sex, clinical cues (evidence of pathology, distress)	Chronic lower back pain	Confirming the hypothesis, physician sex moderates the influence of clinical cues on pain management practices: evidence of pathology had a larger effect on male than on female physicians’ referrals to psychology/psychiatry.
Bories et al, 2018 [56]	To test the hypothesis that physician uncertainty aversion impacts medical decision making for older patients with acute myeloid leukaemia	France	Acute	Physician	Prescription	Age (note clinically relevant)	Physician demographic, occupational, behavioural characteristics	Acute myeloid leukaemia	Physician attitudes to risk influenced chemotherapy decisions for older patients. Physicians opting for intensive chemotherapy (IC) had higher aversion to uncertainty and treated fewer patients annually, than the low IC group but were similar in age, hierarchical status or years of experience.
Burgess et al, 2014 [57]	To test the hypothesis that racial biases in opioid prescribing would be more likely under high levels of cognitive load	USA	Primary care	Physician	Prescription	Ethnicity (black, white)	Physician cognitive load	Chronic low back pain	Hypotheses were partially confirmed. Cognitive load altered ethnic inequalities in prescribing patterns in different ways for male and female physicians. Under high cognitive load, male physicians were more likely to prescribe opioids for White patients; while under low cognitive load, they were more likely to prescribe opioids for Black patients. Female physicians’ bias toward prescribing opioids to Black patients was stronger under greater cognitive load.
Burt et al, 2016 [58]	To examine whether South Asian people rate GP consultations similarly to White British people, in order to understand why minority ethnic groups often give poorer evaluations of primary care	England	Primary care	Public	Consultation style	Ethnicity (South Asian, white)	Patients' ratings of quality	Persistent cough, perforated ear drum, painful elbow generalised numbness	Respondents from a Pakistani background rated communication in simulated GP consultations significantly more positively than their White British counterparts (contrary to the hypothesis that South Asians’ poorer evaluations of primary care experience is due to higher expectations of care).
Daugherty et al, 2017 [59]	To test the hypotheses that physician gender bias would have little effect on treatment decisions for the male patient and would result in lower use of cardiovascular tests among gender-biased physicians for female patients	USA		Physician	Diagnosis	Gender	Implicit bias	Coronary artery disease	Hypotheses were partially confirmed; cardiologists who associated risk taking more with men than with women were more likely to view angiography as useful to diagnose male versus female patients but equally likely to recommend stress testing. Physicians were less certain of diagnosis in women than men.
Elliott et al, 2016 [60]	To test whether hospital-based physicians use different verbal and/or nonverbal communication with black and white simulated patients and their surrogates.	USA	Acute	Physician	Consultation style	Ethnicity (black, white)	Verbal and non-verbal communication between patient & physician	Metastatic gastric and pancreatic cancer	Physicians used similar verbal but different nonverbal communication behaviours with black and white patients.
Fischer et al, 2017 [61]	To test whether patient requests for specific opioid pain medication would lead physicians to classify them as drug-seeking and change management decisions	USA	Primary care	Physician	Prescription	Ethnicity (black, white)	Patient (drug seeking) behaviour	Pain (sciatica)	Physician suspicion of drug-seeking behaviour was much higher when patients requested opioid medication. Physician suspicion of drug-seeking behaviour did not vary by patient characteristics, including gender and race.
Gao et al, 2019 [62]	To test whether Chinese favour family-centred decision making while European Americans favour shared decision making in depression care	USA	Other - mental health	Public	Mode of decision making - hospital or community care	Race, nationality	Acculturation, preferences for care	Depression	Hypotheses were confirmed; Chinese preferred family-centred decision making while Americans preferred shared decision making. Chinese living in America paralleled European Americans.
Green et al, 2007 [63]	To test whether implicit or explicit race biases predict physicians' decisions to give thrombolysis for acute myocardial infarction	USA	Acute and primary care	Physician	Diagnosis, prescription	Ethnicity (black, white)	Physician implicit bias	Acute myocardial infarction	Hypothesis was confirmed. As physicians’ pro-white implicit bias increased, so did their likelihood of treating white patients and not treating black patients with thrombolysis.
Hirsh et al, 2009 [64]	To test whether gendered expectations of pain and facial pain expressions influenced pain assessment and treatment disparities in nurses	USA	Acute	Nurse	Prescription	Age, sex, race	Gender role expectations of pain (sensitivity, endurance, willingness to report), high/low pain facial expression	Pain appendectomy	Hypotheses were partially confirmed; nurses’ gender role expectations of pain didn’t influence decisions but pain expression did. Nurses generally rated female, African American, older patients’ pain higher and were more ready to prescribe opioids.
Johnson-Jennings et al, 2018 [65, 75]	To test whether patient-provider racial concordance and patient ethnic salience is associated with 1) provider pain assessment 2) attitudes toward referral for traditional healing practices for indigenous patients	USA	Primary care	Other clinical professional	1) Prescription 2) referral	Ethnicity (Indigenous American - high/low ethnic salience)	Racial concordance (patient & physician)	Chronic lower back pain	1) Indigenous providers rated patient with higher Indigenous ethnic salience more congruently with the self-reported pain ratings 2) Provider–patient racial concordance increased likelihood of consulting with and referring patients to traditional healing practices.
Lutfey et al, 2009 & 2010 [66, 67]	1) Whether physician certainty is associated with decision making. Explore variations, by health care system, patient characteristics 2) whether observed disparities in CHD decision making are influenced by priming physicians to consider CHD.	USA, Germany and England	Primary care	Physician	Diagnosis, referral, prescription, lifestyle recommendations	Age, gender, ethnicity (black, white), SES	Diagnostic certainty, healthcare system, physician priming	CHD	1) Certainty was positively correlated with test ordering, prescriptions and specialist referrals. Physicians were least certain of CHD diagnoses when patients were younger and female. 2) Primed physicians were more likely to order CHD-related tests and prescriptions than unprimed but main effects for patient, gender and age remained.
Mckinlay et al, 2012 [68]	Whether physicians’ decisions to diagnose diabetes vary by race/ethnicity (after controlling for SES, age, and gender).	USA	Primary care	Physician	Diagnosis	Age, gender, ethnicity (black, Hispanic, white), SES	Effects of SES on ethnicity	Diabetes	Primary care physicians’ vignette diagnosis was patterned by race/ethnicity (rather than by SES). [Undiagnosed signs of T2DM in the community was patterned by SES rather than race/ethnicity.]
Papaleontiou, et al 2017 [69]	Understanding why older thyroid cancer patients are not being referred to high-volume surgeons.	USA	Primary care	Physician	Referral	Age	Physician training, patient volume, discipline & patient preferences	Cancer	Endocrinologists and physicians treating more than 10 thyroid cancer patients per year were more likely to refer older thyroid cancer patients than primary care physicians. Patient preference, transportation barriers and confidence in local surgeon were commonly reported reasons to decrease likelihood of referral.
Samuelsson et al, 2014 [70]	Disentangle a number of determinants on addiction care practitioners' perceptions of the severity of alcohol and drug consumption in clients.	Sweden	Addiction	Other	Referral (eligibility for services), perceptions of severity	Age, gender, ethnicity, SES, family circumstances	% variance due to vignette, professional and work unit	Substance use	Practitioners of different professional backgrounds and workplaces judge alcohol and drug consumption by different norms, and this was also influenced by characteristics of the users.
Shapiro, et al. 2018 [71]	Whether neonatologists show implicit racial and/or socioeconomic biases and whether these are predictive of recommendations at extreme periviability	USA	Acute	Physician	Care: comfort vs intensive (e.g. resuscitation)	Race, SES	Implicit bias	Periviability	Hypotheses were in part confirmed. Physicians with implicit socioeconomic bias were more likely to recommend comfort care to high than low SES vignettes but did not appear influenced by implicit racial bias.
Sheringham et al, 2017 [72]	How patients' clinical and sociodemographic characteristics influence GPs’ decisions to initiate lung cancer investigations	England	Primary care	Physician	Diagnosis	Age, gender, ethnicity (black, South Asian, white), SES	Information elicited, physician attributes	Respiratory symptoms	The information GPs elicited from patient vignettes influenced their decisions but did not explain observed ethnic inequalities in cancer investigations
Tinkler et al, 2018 [73]	Whether appointment offers to new US primary care patients who mention concerns about smoking or weight differ from offers to patients with no health concerns (healthy patients)	USA	Primary care	Other	Appointment offer	Insurance status, race/ethnicity, and gender	Health concerns (smoking/weight concerns vs healthy); state-level Medicaid expansion status	Disease prevention	Patients with smoking concerns were no more likely to be offered new patient appointments than healthy patients and less likely than those with weight concerns. Insurance status influenced access.
Wiltshire,et al. 2018 [74]	Whether concordance leads to higher ratings of trust in physicians amongst African American women race, gender and age	USA	Primary care	Public	Trust	Race - age, gender	Concordance	Breast exam	Older African-American women did not rate race, gender or age-concordant doctors higher on trust; instead they rated white, older male higher on competence than African-American older females.

Key: *SES* socioeconomic status

Most studies (17/21) sought to examine drivers of variations in healthcare in relation to patient ethnicity. Drivers of variation were also examined in relation to patient gender (*n* = 9), socioeconomic circumstances (*n* = 7) and age (n = 9). No studies examined unwarranted variations by other characteristics protected in legislation in some countries, such as disability and sexuality.

### Methodological assessment

We assessed ten studies as being of good methodological quality (Table 3). We focused on these studies in exploring how vignettes may produce insights into drivers of variations of care. Seven studies were assessed as moderate quality, with lower certainty about the insights they could provide into drivers of healthcare variation. Four studies were assessed as low methodological quality, primarily because flaws in their sampling and response rates led to the possibility of significant biases that would compromise the validity of their findings, no matter how well their vignettes were designed and executed. More details on how the 21 included studies were designed and conducted are given below.

**Table 3:** Assessment of included studies according to methodological framework *

Study	Vignette design						Wider study design				Score	Rating
	Credibility	***Number of vignettes***	Variability	***Mode***	Evaluation	Description	Concealment	Realism	Sampling & response	Analysis		
Lutfey 2009 & 2010 [66, 67]	3	*> 1*	1	*Video*	3	1	3	1	5	1	18	**Good**
Samuelsson 2014 [70]	3	*> 1*	1	*Text only*	2	1	2	1	6	1	17	
Adams 2014 [53]	2	*> 1*	1	*Video*	2	1	3	1	5	1	16	
Elliott 2016 [60]	3	*> 1*	1	*Interactive (in-person)*	3	0	1	0	5	1	14	
Tinkler 2018 [73]	2	*1*	1	*Interactive (by phone)*	1	1	3	1	6	n/a	15	
Sheringham 2017 [72]	2	*> 1*	0	*Interactive (online)*	2	1	2	1	4	1	13	
Burt 2016 [58]	3	*> 1*	1	*Video*	3	0	1	0	3	1	12	
Fischer 2017 [61]	3	*1*	1	*Video*	2	0	2	1	2	n/a	11	
Hirsh 2009 [64]	3	*> 1*	1	*Video*	2	1	0	0	2	2	11	
Burgess 2014 [57]	2	*1*	0	*Pictorial*	0	0	1	1	6	n/a	10	
Green 2007 [63]	2	*1*	0	*Pictorial*	2	0	0	1	4	n/a	9	**Moderate**
Daugherty 2017 [59]	2	*1*	0	*Pictorial*	2	1	0	0	4	n/a	9	
Wiltshire 2018 [74]	2	*> 1*	1	*Pictorial*	0	1	0	0	3	1	8	
McKinlay 2012 [68]	2	*1*	0	*Video*	2	0	0	0	4	n/a	8	
Begeer 2008 [54]	1	*> 1*	0	*Text only*	0	0	0	0	5	0	6	
Bories 2018 [56]	1	*> 1*	0	*Text only*	1	1	0	0	2	1	6	
Shapiro 2018 [71]	1	*1*	0	*Pictorial*	0	1	0	0	4	n/a	6	
Johnson-Jennings 2018 [65, 75]	2	*1*	0	*Pictorial*	1	1	0	0	0	n/a	4	**Low**
Bernardes 2013 [55]	2	*>1*	0	*Text only*	0	1	0	0	0	1	4	
Papaleontiou 2017 [69]	1	*> 1*	0	*Text only*	0	0	0	0	2	0	3	
Gao 2019 [62]	0	*1*	0	*Text only*	0	1	0	0	0	n/a	1	

* Scoring system (more detail in supplementary file 2): **credibility** 0-3 (3= construction well described, 2= described to some extent 0/1 = little or no description); ***number*** *(no score);* **variability** 0-1 (1= more than one variant of an experimental factor produced, 0= no); ***mode*** *(no score);* **evaluation** 0-3 (3= well described, 2= described to some extent 0/1 = little or no description); **description** 0-1 (1= full vignette available to view, as much as is practically possible, 0= no); **concealment** 0-3 (3= concealment strategies clearly described or analysis considered effects of awareness, 2= described to some extent, 1 = purpose was not shared but no description of how concealment attempted 0 = no/not stated); **realism** 0-1 (1= attempt to introduce realism into data collection conditions, 0- no); **sampling & response** 0-6 (NB: each score is doubled to account for both sampling and response: 3= random sampling, response & completion rate high, justified exclusions; 2= sample strategy described & justified (purposive or random); response or completion rates fully reported and risk of bias considered; 1= sampling strategy inadequately or not described, inadequate consideration of bias; 0= response rates not given & inadequate consideration of bias); **analysis** 0-2 (2= accounted for clustering & individual/aggregated analysis performed 1= accounted for clustering OR individual/aggregated analysis performed 0= neither n/a = only one vignette shown to participants)

### Vignette design

#### Credibility

Most studies provided comprehensive descriptions of how vignettes were constructed. Higher quality studies described how input from clinicians or patients influenced content and delivery. For example, Burt et al. based vignettes on previously video-recorded patient-clinician encounters [42]. In a three studies, content was based on national guidelines [65, 72, 75].

#### Number

Just over half (12/21) of studies showed participants more than one vignette.

#### Variability

Eight high and one moderate quality study used variants of experimental factors, depicting the same experimental characteristic using more than one actor, photo or video or simulated case.

#### Modality

In six studies, vignette information was purely textual; here, manipulated characteristics and their variations were therefore stated clearly to participants. In 12 studies, vignette information was visual, either pictorial (n=6) or video-based (n=6). Here, manipulated characteristics were communicated non-verbally and may (or may not) have been inferred by the participants. In three studies, vignettes were presented interactively, with one study each using online, telephone and in-person standardised patient approaches. In interactive modalities the content of the vignette could vary across participants, as the vignette evolved in response to respondent behaviours, such as the questions they asked [42].

#### Evaluation

Three high quality studies comprehensively reported how their vignettes performed, most commonly in tests of credibility [58, 60, 68]. Mckinlay et al., Hirsh and Lutfey et al. used post-study quantitative surveys of participants to find out whether vignette 'patients' were typical of the real patients they encountered [64, 66, 68]. McKinlay reported that 91% of participants viewed the vignettes as typical of their patients [68]. Burt et al reported the expert clinical raters’ scores of their high and low performing vignette consultations as an indication of their credibility [58].

Vignettes performance was evaluated in other ways too. For example, Sheringham et al. had developed an online interactive vignettes application specifically for the study [72]. The authors quantified system errors that occurred when the software could not answer a question entered by a participant. System errors occurred on average in just under 5% of all participant interactions. Analysis was adjusted to examine whether system errors could have been responsible for the findings and this was found not to be the case [72]. Description of any kind of vignette evaluation were largely absent from lower quality studies.

#### Description

Thirteen out of 21 studies presented or facilitated access to an entire example vignette. Access to video or interactive vignettes in a journal article is not straightforward, but five out of the nine video or interactive papers did include sufficient aspects (e.g. using video stills [60]) or online links (e.g. to a multimedia demonstration [72]) to enable readers to judge vignettes’ quality and credibility.

### Wider study design

#### Concealment

While eleven papers reported that the study’s purpose was not divulged to participants, only seven high quality described strategies they actively employed to conceal it. These included: stating a wider or different purpose in study information, including a ‘distractor’ (either an unrelated vignette or unrelated tasks during the study), and using free-text response options as opposed to a predetermined selection (*n* = 8) to reduce the risk of priming and response bias.

Three studies illustrated that participants’ awareness of the study purpose could affect the findings [54, 63, 66]. Lutfey et al. (2009) alerted half their sample to the potential of CHD as a diagnosis; primed doctors made different decisions on the same vignettes to those not explicitly primed [66]. Green et al (2007) found a strong relationship between physicians’ implicit bias scores and thrombolysis decisions for black patients in participants unaware of the study’s aim; the relationship was reversed in participants aware of the aim [63]. Finally, ethnic bias in the assessment of autism, found when clinicians’ were asked to give a spontaneous clinical judgement, disappeared when clinicians were asked to specifically rate the likelihood of autism [54].

Active strategies for concealment were not described in any of the low or moderately rated studies.

#### Realism

Six of the moderate and high quality studies sought to collect data in settings that replicated aspects of healthcare delivery, for example by collecting data in physicians’ offices during clinic times [61, 66, 68]. Such data collection was not always achieved as planned; Sheringham et al. sought to conduct an online study in clinic settings between appointments, but due to limited clinic IT facilities many participants completed the study at home [72].

#### Sampling and response rate

Risk of bias was common due to sampling flaws, low or unreported response rates. It was not limited to low quality studies. Eight studies - two higher, two moderate and all the lower quality studies - lacked explanations or justification of sampling selection, recruitment strategy or representativeness of the final sample. Only eight out of 21 studies reported response rates. Of these, three reported response rates of less than 30%. Several studies were unable to report the total population contacted for the study due to the method used to approach participants, such as distribution via clinical networks. Insights were also on occasion limited due to challenges of recruitment. Johnson-Jennings et al. sought to examine the extent to which ethnic concordance between clinician and patient was a driver of variations, but insights were limited as they were only able to recruit 33 Native American physicians [75].

#### Analysis

Where studies presented respondents with more than one vignette, most sought to control for potential effects of a particular depiction by including the vignette as a covariate in multivariable analysis. Appropriate analytical methods were used to account for clustering. Only two studies sought to examine variation between participants: Bories et al. used clustering to identify characteristics of physician behaviour patterns across vignettes, whilst Hirsh et al. analysed decisions at the level of the individual [56, 64]. This individual-level analysis showed that only a minority of nurses displayed non-clinical variations in decisions, but such variations were sufficiently large to influence the aggregate analyses.

### New insights from vignette studies into the drivers of healthcare variations

Studies contributed to understanding variations in care in two ways. Firstly, most of the moderate or high-quality vignette studies (14/17) sought to test specific hypotheses which might explain observed disparities in care – hypotheses which are challenging to examine using real patients. Secondly, studies aimed to provide insights into poorly understood decision-making processes underlying disparities in care. Many papers served both purposes (testing specific hypotheses and providing new insights), with just three focussing only on insights into decision-making [53, 70, 72].

Vignette studies may both lend support to, or challenge, hypotheses for how inequalities in healthcare arise. For example, clinicians frequently make decisions amongst competing demands in chaotic working conditions, which result in a background of high cognitive load, and the potential for subsequent variations in care. It is clear that research assessments of decision-making recorded in quiet environments without time constraints do not replicate this pressure. One study provided evidence that bias is more likely to arise in high pressure situations: increasing cognitive load through the provision of a competing task to do under time pressure altered ethnic inequalities in physicians’ prescribing patterns [57]. This supports not only the notion that cognitive load leads to variations in care, but that such variations may be systematically biased against certain patient characteristics. Of note, additional cognitive load altered inequalities in prescribing in different ways for male and female physician, highlighting the complexity of contextual influences on disparities in care [57].

Two papers used hypotheses generated from real patient data as the basis for tests with parallel vignette studies [54, 68]. Combining insights from observational and vignette data may be particularly helpful in clarifying the relevance of research findings to policy or practice. As an example, in an initial descriptive analysis of case records, Begeer et al. identified that minority ethnic groups were under-represented in autism institutions [54]. In a contemporaneous vignettes study, they found that physicians’ ethnic biases in diagnosing autism disappeared when they were specifically prompted to consider autism. The authors suggest the use of structured prompts in clinical assessments may decrease variations in diagnosis and subsequent care [54].

Whilst such insights lend credibility to prior hypotheses, vignette studies may also bring insights that challenge proposed drivers of reported variations in healthcare. For example, Burt et al. noted that certain minority ethnic groups report lower patient experience scores compared to the majority population across a wide variety of settings [58]. One proposed explanation for this is that minority ethnic patients receive similar care to the majority white patients, but have higher or different expectations of care. To test this hypothesis, Burt et al. presented respondents with video vignettes of GP-patient consultations to gauge their expectations of care. They found South Asian respondents consistently rated GPs’ communication skills higher than white respondents, thus challenging the hypothesis that poorer reported experiences of care in South Asian patients relative to White British patients arise from higher expectations of care [58].

As noted above, vignette approaches may provide insights into decision-making processes. Three studies in this review sought to obtain new insights into how ethnic disparities arise during healthcare encounters. Obtaining generalisable evidence on this is rarely feasible in real life due to the specific dynamics of individual clinician-patient pairs. Adams et al. asked physicians to reflect on video consultations about depression, analysing these narratives in detail to identify micro-components of clinical decision making [53]. This approach, which yielded rich data on the cues physicians reported using and the inferences they drew from them, in fact suggested there was little ethnic bias in physicians’ decision-making processes. Such findings, however, rely on the accuracy of physicians’ retrospectively constructed narratives. More recently, two studies used elements of simulation to explore in real time how interactions between patients and healthcare professionals may lead to variations in care [60, 72]. For example, Elliot et al. coded video recordings of encounters between physicians and standardised patients, and demonstrated that variations in healthcare arose during consultations through differences in non-verbal interactions [60].

Studies within this review were able to test hypotheses and generate new insights into decision-making processes through their deliberate divergence from real life situations, involving the manipulation of vignette characteristics and the contexts in which data were collected. As highlighted by many of the studies above, vignette approaches have been particularly useful to date in examining ethnic disparities in care, with researchers circumventing the obstacles experienced in real life of finding sufficient numbers of patients or clinicians of rare ethnicities to undertake studies in this area. The vignette approach also enables standardisation and isolation of characteristics of interest. Such standardisation helps to eliminate the possibility that observed ethnic variations in healthcare delivery were caused by individuals’ cultural and linguistic, rather than ethnic group, differences.

## Discussion

### Main findings

Experimental vignette studies have been used in a number of innovative ways to examine drivers of unwarranted variations in healthcare delivery. They can test hypotheses proposed to explain variations in care that are not possible using real-life data through the manipulation of vignette characteristics or the context in which data were collected.

By applying a novel methodological framework for conducting vignette studies to this review, we demonstrated that their insights have been limited in many cases by a lack of evaluation of the credibility of vignettes and flaws in their wider study design.

### Strengths and limitations

The volume of literature retrieved from the search for empirical studies was large, and in many cases obviously not relevant to the study question. To manage this volume, we instigated an automated screening process, and used limited double screening. As a result, we may not have captured an exhaustive set of all experimental vignette studies identifying drivers of unwarranted variation in healthcare quality. However, our methods were sufficient for our purposes, which were to identify a set of studies of sufficient quality to illustrate the range of ways in which vignette designs have been used and identify areas in which the potential of vignette methods could be maximised to provide further insights into drivers of unwarranted variations in health care.

There were flaws in almost all of the studies retrieved by our search. In most studies, aggregate analyses of decision making were presented, which may mask heterogeneity between physicians’ (or patients’) perceptions or decision-making behaviour. A number of studies reported findings that were unexpected or counter to findings from observational studies. Without searching discussion about why unexpected findings occurred, such vignette studies may have poor credibility and limited capacity to influence future research or policy. More broadly, studies often had severe limitations in their wider design, notably due to biased or incompletely described samples.

### What this study adds

The application of a novel methodological framework to appraise vignette studies illustrated the variation in quality and conduct of such studies. The framework also adds to existing methodological reviews by consolidating guidance into one source and considering the range of modalities – beyond text and video - that can be used to depict vignette content [42, 43, 45]. This is important because choice of vignette delivery mode determines what research questions it is possible to answer. For example, if a study seeks to examine events during a clinical encounter, static vignette modalities will not capture these [43]. By illustrating the heterogeneity in reporting in this field, it provides evidence of the need for standard reporting guidelines reflecting the full range of possible vignette modalities to enhance the transparency and quality of vignette studies in health services research.

While developed for appraising studies examining drivers of inequalities, it may have wider applicability to assess the methodological rigour of other experimental vignette studies. This is because most dimensions of the framework – namely considerations of vignette credibility, evaluation and description and the wider study design - are central to vignette studies with any purpose. One dimension - the need to conceal the study purpose – may be more specific to inequalities or to studies seeking to examine behaviours or views that participants feel are undesirable. However, we caution against uncritical application of the scoring system developed for this paper. The scores were weighted to reflect the importance of dimensions considered important in this discipline and may well require adaptation for other fields.

## Conclusions

Understanding how unwarranted variations in healthcare arise is challenging. Experimental vignette studies can help with this, but they need careful design and effort to be conducted to a high standard. To date, most experimental vignette studies have concerned themselves with exploring the attitudes and behaviour of healthcare professionals. There is scope for a greater focus on patient attitudes, experiences and behaviours, and the interactions between patients and providers, in determining how variations arise and persist.

The framework developed in this paper to appraise vignette studies covers dimensions of relevance beyond inequalities. Wider application and adaptation is required to determine the extent to which it can ultimately benefit researchers across scientific disciplines.

## Supplementary Information

**Additional file 1.**

## Data Availability

All the data come from peer-reviewed papers listed in the reference list.
