# Supplementary material for: The use of experimental vignette studies to identify drivers of variations in the delivery of health care: a scoping review
Source: BMC Med Res Methodol. 2021 Apr 22;21:81. doi: 10.1186/s12874-021-01247-4 (PMC8061048; doi:10.1186/s12874-021-01247-4)
Supplement: Supplementary file 1 — Additional file 1. [file 12874_2021_1247_MOESM1_ESM.docx]

**The use of experimental vignette studies to identify drivers of variations in the delivery of health care: a scoping review**

**Jessica Sheringham ^1^**

**Isla Kuhn ^2^**

**Jenni Burt ^2^**

**Supplementary file 1 and 2.**

**Supplementary file 1**

**Search strategy (Box 1) and inclusion and exclusion criteria (Table 1)**

### Box 1. Medline search strategy (adapted for other databases)

| ((Vignette* or "paper people" or (case adj (stor* or simulat*)) or scenario* or "policy capture" or "judgement analysis*" or "factorial survey*" or "factorial experiment*" or "virtual patient" or "virtual human" or (simulat* adj patient*)).mp.)and(((variation* or vary* or variet* or inequalit* or unequal* or disparit* or equal* or similar* or dissimilar* or differn* or bia*) AND(Health care or healthcare or health service* or primary care or primarycare or hospital* or community care* or acute or secondary or tertiary or family practi* or general pract* or care)).mp.) |
| --- |

Table 1. Inclusion and exclusion criteria

| Criteria | Included | Excluded |
| --- | --- | --- |
| Timescales | Published 2007 – 2019 | Published before 2007 |
| Health care delivery | Healthcare access, treatment/prescription, referral and diagnosis decisions and communication /consultation styles. | - Clinical trials of interventions to improve effectiveness of health care delivery - studies conducted on students or clinicians in learning environments - studies of clinicians’ knowledge - delivery of social care, other services provided by local government |
| Patient characteristic | Studies examining variations linked to protected characteristics (in England defined under the Equality Act 2010): age, gender, disability, gender reassignment, pregnancy and maternity, race, religion or belief.  Plus: socioeconomic status or circumstances. [1,2] | Studies only focusing on drivers of variation by professional clinical speciality, patient or clinician behaviour, geographic location |
| Vignette methods | Interactive approaches (games, multimedia, fake patient, mystery shopper, simulation)  Video  Text-based (paper, or internet administered surveys) | Descriptions of clinical situations where patient attributes meeting inclusion criteria were not described, e.g. where physicians were given a scan and no other patient characteristics, discrete choice analysis, time trade-off designs |
| Setting | Europe, Canada, USA, Australia, New Zealand | Africa, Asia |
| Publication type | Empirical studies | Reviews, editorials, letters, studies employing solely qualitative analysis techniques, abstracts only |
| Design | Experimental designs: key variables intentionally modified and manipulated e.g. using random allocation, factorial designs | No systematic manipulation of vignette characteristics |
| Purpose | Explanatory: how inequalities in the quality of healthcare delivery may arise. | Descriptive: i.e. purpose is measuring or identifying inequalities. |
| Application of vignette designs or findings | Aims or conclusions relate to behavioural intentions, preferences or attitudes | Aims or conclusions conflate vignette responses with actual healthcare behaviour |

1 Agency of Healthcare Quality. Six Domains of Health Care Quality. http://www.ahrq.gov/talkingquality/measures/six-domains.html (accessed 28 Feb 2020).

2 UK Parliament. Equality Act 2010. Statute Law Database http://www.legislation.gov.uk/ukpga/2010/15/section/4 (accessed 28 Feb 2020).

**Table 2.** Reported funding sources of included studies

| Study | Funding Source |
| --- | --- |
| Adams et al, 2014 | Economic and Social Research Council, grant RES-177-25-0014 |
| Begeer, 2009 | not reported |
| Bernades et al, 2013 | Portuguese Science Funding Agency–Fundac¸ ~ao para a Ci^encia e Tecnologia (PTDC/PSI-PSO/099809/2008). |
| Bories, 2018 | not reported |
| Burgess et al, 2014 | VA Health Services Research & Development: PPO 09–247 (Burgess) and a locally initiated project grant from the Center for Chronic Disease Outcomes Research, Minneapolis VA Medical Center |
| Burt et al, 2016 | National Institute for Health Research Programme Grants for Applied Research (NIHR PGfAR) Programme (RP-PG-0608-10050). |
| Daugherty,et al 2017 | National Heart, Lung, and Blood Institute. Drs Daugherty, Blair and Havranek are currently supported by R01 HL133343 from the National Heart, Lung, and Blood Institute and 15SFDRN24470027 from the American Heart Association |
| Elliott et al, 2016 | Research grants awarded to Dr. Barnato by the American Cancer Society (PEP-08-276-01-PC2) and the National Cancer Institute (R21CA139264). Dr. Elliott was supported by the University of Pittsburgh Medical Center (UPMC) Clinical Scientist Track |
| Fischer et al, 2017 | Award number AR056992 from the National Institute of Arthritis and Musculoskeletal and Skin Disorders (NIAMS) (NIH). The funders had no role in study design, data collection and analysis, decision to publish, or preparation of the manuscript. |
| Gao, 2017 | not reported |
| Green et al, 2007 | Esther B. Kahn Fund of the Department of Emergency Medicine of Brigham and Women’s Hospital. Dr. Green received support from a National Research Service Award, grant no. T32HP11001-15. Dr. Banaji and Dr.Carney received support from the Mind, Brain, and Behavior Initiative at Harvard University and Dr. Banaji received support for the development of the internet-based portion of this study from the National Institute of Mental Health Grant HL-0000013 as well as the Mind Science Foundation. The funding organizations had no role in the design and conduct of the study, including data collection and management, analysis, interpretation of the data, and preparation, review, or approval of the manuscript. |
| Hirsh, 2009 | Grant F31 (NS049675) to A.T. Hirsh from the National Institutes of Health, National Institute of Neurological Disorders and Stroke. Support was also provided, in part, from the National Institutes of Health, National Institute of Child Health and Human Development, National Center for Medical Rehabilitation Research (T32HD007424) |
| Johnson-Jennings et al, 2015 | NIH Grants 5 R01 DA034466 03, L16 Career Award NOT-OD-10-105, and 5R25MH084565 |
| Lutfey et al, 2009 & 2010 | National Institutes of Health, National Institute on Aging (grant #AG16747). The study sponsors had no involvement in the study design; in the collection, analysis, and interpretation of data; in the writing of the report; or in the decision to submit the paper for publication. |
| McKinlay et al, 2012 | Grants from the National Institutes of Health, National Institute of Diabetes, Digestive and Kidney Disorders (Grant Nos. DK66425 and DK056842). |
| Papaleontiou, et al 2016 | University of Michigan Comprehensive Cancer Center IDEA Fund to Dr. Papaleontiou. Dr. Papaleontiou is also funded by the National Institute on Aging of the National Institutes of Health under Award Number K08 AG049684 and the Career Development Pilot Grant from the Cancer Control and Population Sciences Program at the University of Michigan. Dr. Haymart is funded by R01 CA201198 from the National Cancer Institute and by R01HS024512 from the Agency for Healthcare Research and Quality. |
| Samuelsson et al, 2013 | Swedish Council for Working Life and Social Research (FAS) as part of the Centre of Excellence program at SoRAD |
| Shapiro, et al. 2018 | Stefan Bennett Research Fund in Neonatology at NYU School of Medicine |
| Sheringham et al, 2016 | Department of Health Policy Research Programme |
| Tinkler, 2018 | Sarah Tinkler Faculty Enhancement Grant, Portland State University. Rajiv Sharma, National Institutes of Health grant # 1R15MD010224-01, and Incentive Grant, Portland State University. Raven Susu-Mago National Institutes of Health grant #1R15MD010224-01, and Incentive Grant, Portland State University. Miron Stano, National Institutes of Health grant # 1R15MD010224-01. |
| Wiltshire,et al. 2018 | NIH Loan Repayment Program and the Clinical and Translational Science Award (CTSA) program, through the NIH National Center for Advancing Translational Sciences (NCATS), grant UL1TR000427 |

**Supplementary file 2:**

**Report of review of reviews and development of methodological framework for the design and conduct of experimental vignette studies**

**Aim**

The aim of this review was to locate and synthesise previous literature reviews on the use of experimental vignette methods in empirical investigations into the delivery of health care, to inform the development of a methodological framework to appraise the design and conduct of experimental vignette studies.

**Methods**

Search strategy

Articles published between January 2000 and January 2017 were searched using four bibliographic databases: MEDLINE (via Ovid), Embase (via Ovid), Web of Science, and CINAHL (via EBSCO).The search strategy was initially developed in MEDLINE, and adapted for other databases as appropriate. Search terms were developed to encompass all vignette methods applied to studies of medical decision-making, quality improvement, patient safety and variations in health care use, with the addition of a review article search filter adapted from SIGN (see <https://www.sign.ac.uk/search-filters.html>) (Box 1). Forward and backwards citation searches were conducted for each included full text publication.

### Box 1. Medline search strategy (adapted for other databases)

| Vignette*.mp.  AND  (exp Decision Making/ or exp Diagnostic Errors/ or exp Medication Errors/ or exp Medical Errors/ or exp "Quality of Health Care"/ or exp Quality Improvement/ or exp Quality Control/ or exp Quality Indicators, Health Care/ or exp Patient Safety/ or exp Safety Management/ or exp Safety/ or exp Socioeconomic Factors or exp Health Services Accessibility/ or exp Healthcare Disparities/)  AND  (Meta-Analysis as Topic/ or (systematic adj (review$1 or overview$1)).tw. or exp Review Literature as Topic/ or meta analy$.tw. or metaanaly$.tw. or Meta-Analysis/ or Review/) |
| --- |

**Inclusion and exclusion criteria**

We included all publications which reviewed the use of vignette methods in empirical research studies concerned with the quality and safety of, or variations in, health care. We excluded empirical research papers, editorials, letters, and publications not in English.

**Study selection**

Following the removal of duplicate references, JB screened all titles and abstracts against the inclusion and exclusion criteria. A random sample of titles and abstracts (10% of the total) were additionally screened by JS to confirm agreement. Full texts were obtained for all publications with potential relevance; both JB and JS screened these to identify studies for inclusion in the review.

**Data extraction**

Information from each of the included reviews was extracted by JB, covering the study aim, method, search strategy used, number of vignette studies included, how the vignette studies were classified, key results, and any recommendations for or classifications of vignette studies presented. The extracted data were reviewed by JS to confirm completeness.

**Development of framework to appraise the design and conduct of experimental vignette studies**

Recommendations for the conduct of vignette studies identified within located reviews were examined and extracted. To ensure methodological developments from outside of the health care field were considered, we conducted a further search to identify additional reviews and commentaries with particular relevance for the development of quality criteria. Using forward and backward citation searching, we traced the field of experimental vignette research in disciplines including organisational research, applied psychology, social research, and educational research. Recommendations for the conduct of experimental vignette studies were extracted and added to those made within the health care literature.

**Results**

**Review selection**

Database searches identified 482 citations; following de-duplication, 423 citations were left for title and abstract screening. Of these, 11 full text publications were obtained to review in detail, of which 6 met the inclusion criteria for this review. [1–6] An additional review, which was eligible for inclusion, was located through forward citation searches [7] (see PRISMA flowchart, Figure 1).

Full text articles excluded (n = 5)

(not reviews of vignette methods in health care)

References identified through databases (n=482)

- 176 – Medline
- 107 – Embase
- 181 – Web of Science
- 18 - CINAHL

Reviews included in review

(n = 7)

Full text located from citation search (n=1)

Full texts for screening

(n = 11)

Titles/abstracts excluded (n = 412)

Duplicates excluded (n = 59)

Titles/abstracts for screening

(n = 423)

Figure 1. PRISMA flowchart

**Review characteristics**

The majority of reviews focussed on the use of vignettes in a particular research area of interest, commonly clinical decision-making [2,3,6,7] or doctor-patient communication [4,5]. with one review providing a narrative of the use of vignettes in general in the health care field [1] (see Table 1). With the exception of Hughes, [1] reviews were concerned with one vignette modality and/or type of respondent; written vignettes for clinicians [2,3,6,7]; vignettes for ‘analogue patients’ [4]; or video vignettes [5]. Concerns were raised over the conduct and reporting of empirical vignette studies to date [3,5]. The development of overly complex vignettes, placing too high a cognitive burden on respondents, along with poorly designed analytical approaches were particular concerns identified in the conduct of many studies of clinical decision-making [3]. Additionally, the approach used in many studies to develop and test vignettes was often poorly reported [5], reducing their perceived credibility. Many reviews called for further methodological developments in the field [3–6]. Additionally, a wide range of recommendations for designing vignettes and vignette studies were given [2–7]; however, these were typically confined to one vignette approach only, and we found no review that gave recommendations for the design and conduct of vignette studies across a range of research topics and vignette modalities.

**Table 1.** Description of included reviews

| Author | Year | Method | Area of focus | Key findings | Conclusions | Notes |
| --- | --- | --- | --- | --- | --- | --- |
| Hughes [1] | 2002 | Review | Health care attitudes, perceptions and beliefs | Narrative article considers the distinction between vignettes and real-life processes, and the practical advantages and pitfalls of vignette studies in general | Highlights issues of external validity, as vignettes cannot ever hope to "capture the reality of people's lives"; the generalizability of vignette studies must be carefully considered | Review focus is on uses and interpretation across all vignette modes |
| Veloski [2] | 2005 | Review | Clinical decision-making and practices | Outlines three particular considerations in vignette-based studies: (1) the need for unambiguous instructions; (2) the portrayal of realistic clinical situations; and (3) the need to avoid looking at individual physician responses in the analysis - argues that vignettes should only be used when physicians' are reassured that aggregate responses are to be used, and they are not being "judged" in the way they may be in an assessment setting | Argues for the use of vignettes to assess clinicians’ behaviours | Review focus is on written vignettes aimed at clinicians only |
| Bachmann [3] | 2008 | Systematic review | Clinical decision-making and practices | 27/30 studies looked at decision making of medical experts, covering 28 different medical problems. The median number of attributes manipulated within vignettes was 6.5 (range 2-15); the median number of vignettes used in each study was 25 (range 3 to 130). Five studies used patient files to construct the vignettes; 12 studies used the literature; 7 expert opinion; 1 guidelines. | Raised concerns over the design of vignette studies to date in relation to (a) cognitive burden, with the use of too many attributes and vignettes; (b) a lack of use of normative benchmarks to externally assess opinions expressed, and their deviations from agreed norms (3) poor accounting for correlated data in analyses | Review focus is on written vignettes aimed at clinicians only |
| van Vliet [4] | 2012 | Systematic review | Doctor-patient communication | Identifies general, ethical, and methodological rationales for conducting video vignette studies. In studies reviewed, internal validity was sought by ensuring manipulations were "successful" (involving analogue patients or experts in the script production; or using content from clinical interactions). Only three studies used numerical data to consider whether the manipulations had resulted in varying communication between vignettes. Suggests external validity may be checked by measuring how far analogue patients "identified" with the vignette patient; whether the analogue patients thought the video was "credible"; and by approaches such as only showing the physician in the vignette. | The methodological soundness of scripted video-vignette studies may be increased by providing a specific rationale and increasing internal and external validity. Argues that further research is required into comparisons between analogue and actual patient ratings | Review focus is on vignettes using 'analogue patients' to assess doctor-patient communication only |
| Hillen [5] | 2013 | Review | Doctor-patient communication | Recommends a five-phase approach to developing video vignettes: (1) deciding if video vignettes are appropriate; (2) developing a valid script; (3) designing valid manipulations; (4) converting the scripted consultations to video; (5) administering the videos | Argues that there are still limited methodological developments and understanding in use of video vignettes, and that consequently many published studies do not fully explain the processes used. | Review focus is on video vignettes only |
| Evans [7] | 2015 | Review | Clinical decision-making and practices | Sets out the importance of construct, internal, and external validity, outlining the need for realistic vignettes to differentiate between clinical opinion and be generalizable to 'real-world' settings. Presents recommendations for the content of vignettes, drawing on examples from case-controlled vignette field studies examining clinician's responses to ICD-11 versus ICD-10 classifications | Argues that well-designed vignettes can combine strengths of both survey and experimental methods, and provide generalizability to 'real-life' clinical judgments and decision-making | Review focus is on written vignettes aimed at clinicians only |
| Converse [6] | 2015 | Review | Clinical decision-making and practices | Outlines three "decision points" to consider when designing clinical vignette surveys: (1) question type (open or closed); (2) question format (dichotomous/multiple choice/Likert scale/fill in the blank with numeric response); (3) mode of administration (paper/telephone or in person/computer). Also outlines other design considerations, including (1) selecting decisions to study; (2) realism; (3) establishing validity; (4) pre-testing; (5) administration | Sets out the case for clinical vignettes having advantages over case notes review or standardised patients, but notes that further research is needed into the use of paper-based, closed-ended vignette surveys | Review focus is on written vignettes aimed at clinicians only |

**The development of quality criteria for experimental vignette studies**

Bringing together recommendations from within the health care field with those from the broader vignette literature, we developed a set of ten considerations to assess the design and conduct of experimental vignette studies (Table 2). We subsequently developed a scoring system to enable an overall judgement of certainty to be made about the methodological robustness of experimental vignette studies included in the main empirical paper review – see Table 3 for details.

**Table 2.** Methodological framework for assessment of experimental vignette studies

| 1. Vignette design | |
| --- | --- |
| 1. Credibility | - The degree to which vignettes credibly represent critical aspects of a clinical scenario or patient to potential participants is crucial to the success of an experimental vignette study. [30] - Lens model approaches (studies which compare optimal versus actual decisions in a given situation, originally developed by Brunswick in 1950) have demonstrated empirically that the decision-making performance of participants is improved when situations are realistic. [36] - Basing vignettes on real-life data, clinical expertise, and existing guidelines are recommended ways of enhancing credibility. [19,33,37] |
| 1. Number | - Presenting participants with more than one vignette enables examination of variations in judgement within individuals as well as between them – that is, the extent to which each participant is differentially influenced by each experimental factor in making their decisions. - For example, in vignette approaches based on the lens model, [38] it is typically recommended that there are at least five different representations for each experimental factor. - Depending on the aims and design of the study, the order in which vignettes are presented may need careful consideration. |
| 1. Variability | - Developing or using a number of different representations of each experimental factor may increase study generalisability, by reducing the possibility that idiosyncrasies in one particular representation are responsible for findings. For example, using one female and one male actor in video vignettes may lead not to participants responding to the constructs of gender, but to *that* particular female or *that* particular male. - Where participants do view more than one vignette, analysis must account for clustering of vignettes by respondent, to avoid over-estimating the statistical significance of any effect. [39] |
| 1. Mode | - The mode through which vignettes are delivered has an important influence on the research question an experimental vignette study can answer. - Vignette mode has historically been textual only, with participants presented with a written scenario. Text-based vignettes may constrain not just the information the respondent is given, but how this information is framed. - More recently the use of pictures, videos, actors, and interactive environments have been developed. [15,33] - Pictorial modes are particularly suited to examination of characteristics, such as ethnicity, where visual representation removes the need for explicit statement (and prior framing) of the characteristic. - Studies using video vignettes extend this still further by enabling participants to form judgements on body language and speech patterns in addition to visual cues. - Interactive formats, such as unannounced standardised patients or virtual reality set-ups, have the potential to mimic real delivery which enables exploration of how inequalities may unfold *during* a clinical encounter, through enabling explorations of variations in the information that clinical participants elicit from patients or in both parties’ non-verbal communication. Such approaches are more complex to construct and more costly to develop than static vignette formats, which may limit their feasibility. |
| 1. Evaluation | - Evaluation of vignettes’ face validity – during vignette construction and once data are collected – is key to understanding the validity of findings in studies using vignettes. - Thinking through in advance what is needed to make particular vignettes ‘successful’ for their target audience will guide the nature of and approach to evaluation. - Options include assessment by an expert panel, feedback from participants, or comparing responses to the vignettes to an additional data source such as clinical data.[19,33] |
| 1. Description | - Readers of vignette study papers need to be able to form their own judgments of vignette credibility. An entire vignette should be provided to enable them to do so. |
| 1. Wider study design | |
| 1. Concealment | - When investigating unwarranted variations in care, it is important to conceal the purpose of such studies, given that few people will volunteer behaviours or attitudes that they recognise as poor or biased. - If the study’s purpose is not adequately masked it can bias results, even with carefully constructed vignettes. [24] Participants may learn of the study purpose directly (from study information shared at recruitment) but also may infer it indirectly, through other cues in study materials (e.g. funder’s name), or pre-specified responses that prime participants to consider certain answers. |
| 1. Realism | - External validity of vignette studies is enhanced when studies are conducted in a setting as close as possible to “the natural ecology of decision-making”. [35] - The generalisability of studies to investigate unwarranted variation in healthcare may be improved by collecting data in a setting that mimics key aspects of clinical settings, whether that be the actual environment, other inclusion of features such as the imposition of time constraints. |
| 1. Sampling & response | - The representativeness of any survey rests on sampling, coverage, and nonresponse. - This is particularly important for studies of healthcare variations, where a biased sample or responses – for physician or patient participants – may lead to over- or under-estimation of variations. - Studies need to justify their sample design, sample size, approach to recruitment, response and completion rates, and reasons for excluding data. [40] - The implications of low or biased responses should be considered. |
| 1. Analysis | - Experimental vignette studies are often complex in how data are structured. Analysis must appropriately account for hierarchies within the data. [15] |

**Table 3.** Methodological framework and scoring system

| Methodological consideration | Scale | Score |  |
| --- | --- | --- | --- |
| 1. Vignette design | | |  |
| 1. Credibility | 3= construction well described, 2= described to some extent 0/1 = little or no description | 0-3 |  |
| 1. Number | More than one vignette shown to participants, only one vignette shown to participants | no score - accounted for in analysis |  |
| 1. Variability | Yes - more than one variant of an experimental factor produced (1), No (0) | 0-1 |  |
| 1. Mode | Text only; Pictoral or Video; Interactive (in-person, online, phone) | no score |  |
| 1. Evaluation | 3= well described, 2= described to some extent 0/1 = little or no description | 0-3 |  |
| 1. Description | Yes - full vignette available to view, as much as is practically possible (1); no (0) | 0-1 |  |
| 1. Wider study design | |  |  |
| 1. Concealment | 3= concealment strategies clearly described or analysis considered effects of awareness, 2= described to some extent, 1 = purpose was not shared but no description of how concealment attempted 0 = no/not stated | 0-3 |  |
| 1. Realism | yes - attempt to introduce realism into data collection conditions (1); no attempt (0) | 0-1 |  |
| 1. Sampling & response | 3 = random sampling, response & completion rate high, justified exclusions; 2 = sample strategy described & justified (purposive or random); response or completion rates fully reported and risk of bias considered; 1 = sampling strategy inadequately or not described, inadequate consideration of bias;  0 = response rates not given & inadequate consideration of bias | 0-6  (NB – each score is doubled to account for both response and sampling) |  |
| 1. Analysis | 2 = accounted for clustering & individual/aggregated analysis performed 1 = accounted for clustering OR individual/aggregated analysis performed 0 = neither n/a = only one vignette shown to participants | 0-2 |  |

**References**

1 Hughes R, Huby M. The application of vignettes in social and nursing research. *J Adv Nurs* 2002;**37**:382–386.

2 Veloski J, Tai S, Evans AS, *et al.* Clinical Vignette-Based Surveys: A Tool for Assessing Physician Practice Variation. *Am J Med Qual* 2005;**20**:151–7. doi:10.1177/1062860605274520

3 Bachmann LM, Mühleisen A, Bock A, *et al.* Vignette studies of medical choice and judgement to study caregivers’ medical decision behaviour: systematic review. *BMC Med Res Methodol* 2008;**8**:50. doi:10.1186/1471-2288-8-50

4 van Vliet LM, Hillen MA, van der Wall E, *et al.* How to create and administer scripted video-vignettes in an experimental study on disclosure of a palliative breast cancer diagnosis. *Patient Educ Couns* 2013;**91**:56–64. doi:10.1016/j.pec.2012.10.017

5 Hillen MA, van Vliet LM, de Haes HCJM, *et al.* Developing and administering scripted video vignettes for experimental research of patient-provider communication. *Patient Educ Couns* 2013;**91**:295–309. doi:10.1016/j.pec.2013.01.020

6 Converse L, Barrett K, Rich E, *et al.* Methods of Observing Variations in Physicians’ Decisions: The Opportunities of Clinical Vignettes. *J Gen Intern Med* 2015;**30**:586–94. doi:10.1007/s11606-015-3365-8

7 Evans SC, Roberts MC, Keeley JW, *et al.* Vignette methodologies for studying clinicians’ decision-making: Validity, utility, and application in ICD-11 field studies. *Int J Clin Health Psychol* 2015;**15**:160–70. doi:10.1016/j.ijchp.2014.12.001
